# Recent Advances of Strategies and Applications in Aptamer-Combined Metal Nanocluster Biosensing Systems

**DOI:** 10.3390/bios14120625

**Published:** 2024-12-18

**Authors:** Ki-Beom Kim, Sang-Ho Kim, Seung-Min Yoo

**Affiliations:** School of Integrative Engineering, Chung-Ang University, Seoul 06974, Republic of Korea; plus1215@cau.ac.kr (K.-B.K.); if_only0421@naver.com (S.-H.K.)

**Keywords:** DNA, aptamer, metal nanocluster, detection, biosensor, DNA template

## Abstract

Metal nanoclusters (NCs) are promising alternatives to organic dyes and quantum dots. These NCs exhibit unique physical and chemical properties, such as fluorescence, chirality, magnetism and catalysis, which contribute to significant advancements in biosensing, biomedical diagnostics and therapy. Through adjustments in composition, size, chemical environments and surface ligands, it is possible to create NCs with tunable optoelectronic and catalytic activity. This review focuses on the integration of aptamers with metal NCs, detailing molecular detection strategies that utilise the effect of aptamers on optical signal emission of metal NC-based biosensing systems. This review also highlights recent advancements in biosensing and biomedical applications, as well as illustrative case studies. To conclude, the strengths, limitations, current challenges and prospects for metal NC-based systems were examined.

## 1. Introduction

Biosensors that incorporate various nanomaterials provide numerous benefits, such as low limits of detection (LoD), real-time analysis and the capability for multiplex detection [1,2,3]. Advances in novel nanomaterials have spurred the development of diverse sensing nanoplatforms, physiochemical methods, bioconjugation techniques and binding ligands, leading to innovative strategies that improve detection performance [4]. These biosensors have been developed using diverse nanomaterials, including nanoparticles (NPs), nanorods, nanowires and quantum dots (QDs), along with carbon-based materials, such as carbon dots (CDs), carbon nanotubes (CNTs) and graphene oxide (GOx) [5,6,7,8,9,10].

Metal nanoclusters (NCs) are promising alternatives for organic dyes and QDs, as they exhibit special physical and chemical properties, including fluorescence, chirality, magnetism and catalysis, which contribute to achievements in many fields, including biosensing, biomedical diagnostics and therapy [11,12,13]. By adjusting their components, size, chemical environments and surface ligands, it is possible to create diverse NCs with unique fluorescence properties that can be tuned to different wavelengths in the visible and near-infrared (NIR) spectra. Metal atoms can aggregate in response to reduction agents and form strong covalent interactions with various ligands, such as amino groups, sulfhydryl groups and phosphorus groups found in thiol compounds, dendrimers, polymers, polypeptides and proteins. In recent years, DNA has been explored as a template for metal NCs, thereby increasing their utility [13].

The implementation of functional DNAs, such as aptamers, can endow metal NC-based biosensing systems with specific recognition functions. Aptamers are short strands of single-stranded DNA (ssDNA), single-stranded RNA (ssRNA) or synthetic nucleic acids (XNAs) that can selectively bind to a diverse range of targets, including small molecules, peptides, proteins and even entire cells [14,15,16]. They offer numerous advantages, including high affinity, high specificity, remarkable thermostability, resistance to acidic and basic conditions, low immunogenicity, low toxicity, cost-effectiveness, ease of synthesis and ease of modification [17]. These advantageous properties have contributed to an increase in their utilisation as bio-recognition ligands in a variety of industrial, environmental and clinical applications. Additionally, the growing use of aptamers has prompted the development of innovative strategies and technologies for their discovery, alongside the emergence of several companies focused on aptamer research and synthesis [14].

**Figure 1:**
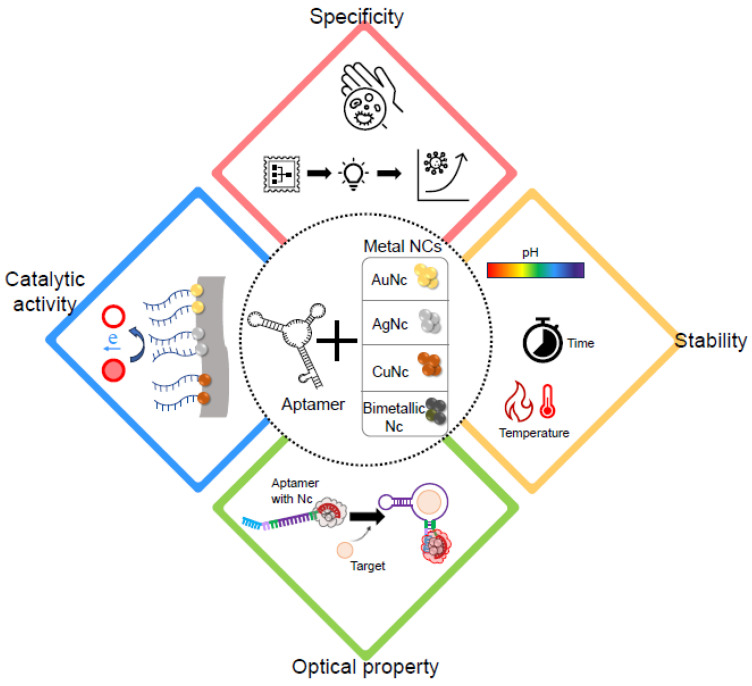
Schematic illustration highlighting the features of aptamer and metal nanoclusters.

In recent years, several reviews provide detailed information on the principles and characteristics of metal NC biosensors [11,18,19,20,21,22,23,24]. This article focuses on systems that integrate aptamers with metal NCs (Figure 1), exploring the advantages and potential applications of such systems. It also introduces molecular detection strategies and technologies, highlighting how the aptamer influences the optoelectronic properties of the metal NC-based biosensing system by modulating the properties of the metal NCs. These sensing strategies can be largely divided into the following categories: (1) the signal changes based on aptamers bound to NCs, (2) the signal changes based on aptamers fused with DNA templates for NC nucleation and (3) the signal changes based on aptamers hybridised with templates for linking to NCs. The advantages and disadvantages of diverse metal NCs are described in this review. To provide further understanding and insights, recently developed techniques in biosensing, biomedical diagnosis and therapy are introduced. Based on these sensing strategies, an overview of representative applications of DNA-templated metal NC systems that use aptamers is described. These applications include examples described in this review, along with others developed for detecting various molecules. Additionally, a critical evaluation of the strengths and drawbacks of these techniques is beneficial for all researchers in both academia and industry involved in the development of metal NC-based systems. Finally, we outlined current challenges and future perspectives. The review will facilitate a more profound comprehension and insights into the prospective applications and attributes of systems that integrate aptamers with NCs, thereby extending the knowledge base established by existing reviews on NCs.

## 2. Metal NC Aptasensor for Molecule Detection and Monitoring

### 2.1. Metal NC as a Sensing Material

Metal NCs are nanomaterials that are smaller than 2 nm and typically comprise up to 150 metal atoms, a size range comparable to the Fermi wavelength of an electron [12]. In this range, the electronic structure undergoes a transition from continuous energy bands, which are characteristic of bulk metals, to discrete energy levels that are analogous to those observed in molecules [25,26]. This size-induced quantum effect gives rise to distinctive optical absorption characteristics and the formation of well-defined HOMO-LUMO gaps [27,28]. Additionally, the facile synthesis process, typically characterised by uniform size and minimal complexity, provides a notable advantage. These NCs exhibit a large Stokes shift and good luminescence stability. Also, the fluorescence of metal NCs can be customised from visible to NIR light wavelengths, facilitating advancements in biomedical applications, including biosensing, bioimaging, biomedical diagnostics and biomedical therapy [29]. Fluorescence modulation can be achieved by utilising various metals, including gold (Au), silver (Ag), copper (Cu) and bimetallic NCs. Employing surface ligands is another fluorescence emission-tuning strategy.

While most metal NCs are synthesised via direct reduction, involving the conversion of metal ions into atom particles using reactive substances, this method can often result in issues, such as weak fluorescence, low quantum yield and susceptibility to aggregation. The incorporation of surface ligands in metal NCs can enhance stability, nanocrystalline nucleation rate and fluorescence by altering the size, electronic structure and optical properties of the NCs [30,31,32,33,34,35,36,37,38,39,40]. The robust interaction between the metal core and surface ligands markedly affects the electronic and photophysical properties, thereby enabling highly efficient photoluminescence. The precise arrangement of metal atoms and ligands creates unique electronic transitions and emission pathways, which are highly sensitive to external factors such as core size, ligand chemistry, aggregation state and surrounding environment [41,42,43]. These characteristics not only define their fluorescence and optoelectronic properties but also allow their emission intensity and wavelength to be tuned, supporting versatile applications in sensing, bioimaging and advanced optoelectronic systems [44]. Additionally, DNAs can serve as essential templates or scaffolds for metal NC synthesis by effectively binding metal ions. The DNA bases anchor and promote the growth of the NCs. Their nano-sized and robust geometric structure, along with their programmable properties, which are based on length, base and conformational state, facilitate the construction of nanostructures with diverse morphologies [44]. Soluble NCs enhance biosensing capabilities, enabling detection in real samples, while their fluorescence allows for the development of innovative probes that surpass traditional fluorophores. Furthermore, a tunable fluorescence property and high photostability can be achieved by varying the bases that alter the sequence or structure of metal NCs. This customisation allows for fluorescence adjustments, facilitating the generation of multiple colours for multifunctional and simultaneous monitoring [45,46]. In addition, DNA-templated metal NCs exhibit minimal effects on cell viability.

### 2.2. Diverse Metal NCs for the Detection of Molecules

A wide variety of NCs can be produced using metal materials beyond the conventional Au and Ag, including Cu and bimetals. Each metal NC exhibits unique properties, such as distinct emission spectra and catalytic activities (Table 1).

Au is a commonly used metal because of its biocompatibility and well-established chemistry. AuNCs exhibit strong fluorescence and are used in bioimaging, sensing and therapeutic applications. They exhibit lower toxicity than many other metals, making them suitable for biomedical applications. Additionally, AuNCs demonstrate strong and size-tunable fluorescence, which allows for customisation in different settings. Their reduced susceptibility to oxidation ensures consistent performance, further contributing to their reliability. Specifically tailored DNA-AuNCs can form stable NCs with unique fluorescence properties. Moreover, AuNCs serve as electrochemiluminescent (ECL) luminophores with tunable luminescence properties [47].

AgNCs are widely accepted NCs owing to their unique properties, including strong fluorescence and potential antimicrobial effects [48]. They have been extensively studied for their formation through the controlled reduction of metal ions on preselected nucleic acid templates, with variable sequence combinations. Ag^+^ ions primarily interact with C-rich sequences or similar ssDNA to form these NCs [49,50,51]. Alternative structures of ssDNA, such as hairpin or dumbbell-shaped DNA with loops, mismatched dsDNA and dsDNA with abasic sites that has neither a purine nor a pyrimidine base, have been used as templates for synthesising AgNCs [52,53,54,55]. AgNCs exhibit a broad emission spectrum, ranging from blue/green to NIR, allowing for in vivo deep tissue imaging [46,56,57]. Additionally, AgNCs can enhance electrochemical signals, enabling ultra-sensitive quantification in various applications [58]. The introduction of diverse quenchers, such as cysteine [59], GOx [60], molybdenum carbide (Mo_2_C) nanotubes [61], carbon nanoparticle oxide (CNPs) [62], tungsten disulfide (WS_2_) nanosheets [63] and molybdenum disulfide (MoS_2_) nanosheets [64], enables a turn-off and label-free approach with strong anti-inference ability and high NC sensitivity when used on complex biosample assays. Another distinct characteristic of AgNCs is their antibacterial activity [65]. The DNA scaffold sequence used for nucleation can affect the antibacterial activity of NCs [66]. The utilisation of scaffolds, such as branched DNA, has been demonstrated to enhance the contact area between NPs and bacteria, thereby promoting antibacterial activity [67,68].

CuNCs have demonstrated potential for developing a simple, cost-effective and label-free fluorescence biosensing system owing to the abundance and affordability of Cu compared to other noble metals, such as Au and Ag. The presence of poly-T bases is essential for the formation of CuNCs. These NCs can be easily synthesised in the presence of specific dsDNA or poly-T base DNA and a reducing agent (such as ascorbic acid) at room temperature. The resulting CuNCs emit a red fluorescence with a long Stokes shift, which is advantageous for minimizing interference from background signals in biological systems [69,70,71,72,73]. High fluorescence emission and photostability have been observed in DNA templates with prolonged chains and a high number of AT-rich sequences [70]. The stability of these templates at high salt concentrations makes them suitable for biosensing strategies based on dsDNA formation reactions [73,74,75,76]. The CuNCs offer an additional advantage for in vivo applications due to the existence of cellular and molecular mechanisms that govern the uptake and excretion of Cu [61].

Bimetallic NCs comprise different metals stabilised and templated by DNA. This combination of metals can lead to unique optical and electronic properties that are not present in single-metal systems. Bimetallic NCs provide enhanced optical and catalytic properties, increased stability due to the synergistic effects of the metals and customisable functions through adjustments in metal composition and ratios. However, they also have limitations, including susceptibility to oxidation, a restricted range of fluorescence colours and relatively low overall stability.

### 2.3. Strategy for Molecule Detection Using Metal NC Aptasensors

**Figure 2:**
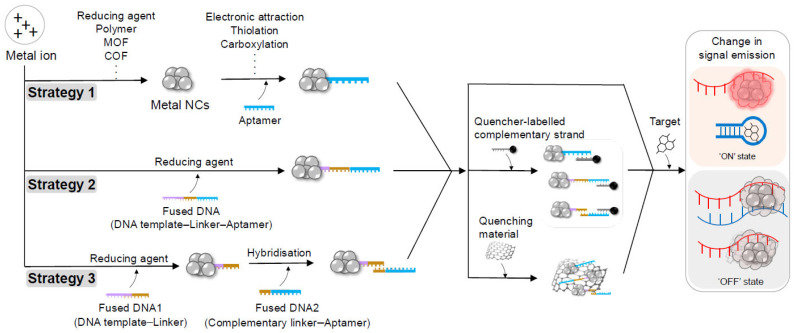
Schematic illustration of three strategies for signal changes induced by combining aptamer with metal NCs. MOF, metal-organic framework; COF, covalent-organic framework.

The combination of aptamers with metal NCs provides specific recognition functions and the enhanced stability of metal NCs, which modulate the electronic structure and steric environment of the NCs [77,78,79]. The binding of target molecules to the aptamer induces conformational changes in DNA, thereby altering the arrangement of metal atoms and subsequently changing the fluorescence intensity, emission wavelength or catalytic activity. In metal NC-based aptasensors, several systems can induce diverse optoelectronic outputs by altering the properties of the NC-based sensing system (Figure 2). The first of these systems involves the direct binding of the aptamer to the NC [47,63,80,81,82,83,84,85,86,87,88,89,90,91,92,93,94,95,96]. Metal ions are typically nucleated by adding reducing agents or polymer scaffolds, such as polyethyleneimine (PEI), and the surface of the resulting NCs can be functionalised with aptamers through mechanisms, such as electrostatic attraction [95], thiolation [94] and carboxylation [80], resulting in changes in fluorescence or catalytic activity. These aptamer-functionalised NCs can additionally bind to a quencher via van der Waals interaction, causing fluorescence resonance energy transfer (FRET) due to the proximity of the NCs (fluorescence donor) and the quencher (fluorescence acceptor); this could lead to reduced fluorescence [80,94]. Recently, significant attention has been directed towards the modification of metal NCs by incorporating cages, such as polymers, metal-organic frameworks (MOFs) and covalent-organic frameworks (COFs), and functionalising diverse polymers. These strategies can enhance the properties of NCs; however, they typically involve complex processes and are associated with high costs.

The second system involves the fusion of aptamers with a DNA template for NC nucleation [59,64,65,73,97,98,99,100,101,102,103,104,105]. This system typically involves the use of a scaffold comprising an aptamer for target binding, an additional domain and a template for NC nucleation. The additional domain can serve as a signal enhancer (e.g., G-rich overhang for AgNCs) [100] or as a sequence for target-cycling strand displacement amplification [73]. The binding interaction between the aptamer and target molecule can either maintain the fluorescence of the NCs through sandwich hybridisation with another aptamer attached to the surface of the material [101,102] or induce a conformational change in the scaffold, thereby resulting in enhancements or reductions in fluorescence emission [73]. Another strategy involves the use of a quencher, where there is competition between the target and the quencher, leading to a fluorescence emission switch from a ‘turn-on’ to a ‘turn-off’ mode [64]. This strategy involves altering both the specific recognition capability of the aptamer and the optical properties of the NCs, leading to challenges in precisely controlling the optoelectronic properties when using the integrated scaffold of aptamer and signal unit.

The third system is based on hybridising the aptamer with a template to link to the NC [76,106,107,108,109]. Hybridisation-induced signal switching systems require two strands: one is a template-extending strand containing a sequence for NC formation and a sequence partially complementary to the aptamer strand, and the other is an aptamer strand that can serve as quencher [106]—and a signal transducer [108]. The NCs are generated by the template containing a region complementary to the aptamer, which can be regulated by the aptamer via hybridisation, thereby resulting in a fluorescence shift. The aptamer strand can also be functionalised with a quencher molecule or other sensing labels to ensure dual-mode detection [109]. In contrast to the two previously discussed systems, this third approach involves a more intricate process. However, it offers greater flexibility in signal control by adjusting the lengths and quantities of complementary strands.

## 3. Current Aptamer-Combined Metal NC for Chemical and Biomolecule Detection

In this section, we introduce a chemical and biomolecule detection system based on various strategies. The challenges and strategies to improve the performance of colourimetric sensing systems, including the related parameters (usage, analyte, linear range, LoD, etc.), are also summarised in the Tables below.

### 3.1. Signal Changes by Aptamer-Functionalised NCs

Aptamers can bind directly to the surface of NCs [47,63,80,81,82,83,84,85,86,87,88,89,90,91,92,93,94,95,96]. After nucleation of metal ions with the addition of reduction agents, such as NaBH_4_ and NaOH, the aptamer can either electrostatically adsorb onto the surface of NCs or attach through functional groups. This adsorption or attachment induces a change in catalytic activity or fluorescence of the NCs. Subsequently, aptamer-target binding restores catalytic activity or fluorescence. 

For instance, a CuNC-based aptasensor was developed for the detection of oxytetracycline (OTC) [95]. The OTC-specific aptamer reduced CuNC catalytic activity and Raman signals by adsorption. Upon OTC binding, the aptamer detached, restoring activity and promoting AuNP formation, enhancing Raman signals. The system achieved LoDs of 18.0 ng (SERS) and 25.0 ng/L (RRS), with linear ranges of 37.5–300 ng/L (SERS) and 37.5–225 ng/L (RRS).

**Figure 3:**
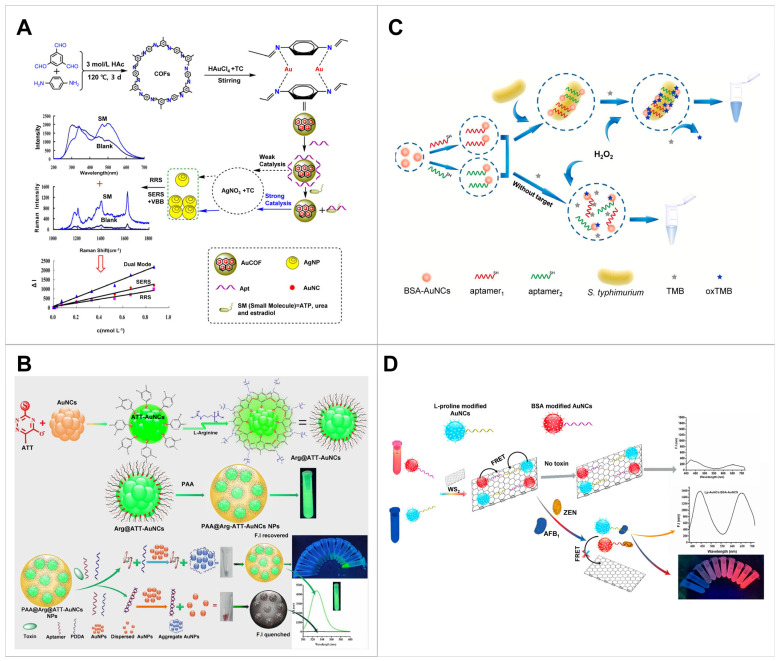
Sensing strategy based on signal emission changes in aptamer-linked metal NCs. (**A**) Detection of urea, ATP, estradiol using NC-loaded COF and aptamer. This system is dual-mode SERS and RRS sensor. Reproduced with permission from [93]. Copyright 2020, Elsevier. (**B**) Detection of T-2 toxin using PAA@Arg@ATT-AuNCs NPs and aptamer–PDDA complex. This system used FRET between PAA@Arg@ATT-AuNCs (fluorescence donor) and AuNPs (energy receptor). Reproduced with permission from [86]. Copyright 2020, Elsevier. (**C**) Detection of *Salmonella typhimurium* using AuNCs@aptamer and TMB. This system enables simultaneous binding of bacteria to both the aptamer@AuNCs and TMB, facilitating peroxidase-like activity due to the increased proximity of these interactions. Reproduced with permission from [91]. Copyright 2020, Elsevier. (**D**) Detection of two different mycotoxins (aflatoxin B1 and zearalenone) using FRET between the AuNCs and WS_2_ quencher. Reproduced with permission from [63]. Copyright 2019, American Chemical Society. NC, nanocluster; ATP, adenosine triphosphate; COF, covalent-organic framework; PAA, polyacrylic acid; ATT, 6-aza-2-thiothymine; PDDA, poly (diallyldimethylammonium chloride); TMB, tetramethylbenzidine.

This strategy enhanced AuNC properties by incorporating them into COFs, which are porous, lightweight and stable [110,111]. The AuNCs loaded onto the COF bonded weakly to the aptamer, thereby reducing the catalytic activity of the NCs [93] (Figure 3A). Upon the introduction of the target molecule, the aptamers were released from the NC-loaded COF by binding with the target. This release triggered a strong catalytic response, thereby facilitating the production of AuNPs [83,93]. The incorporation of a COF enhanced the sensing performance (LoDs of 0.07 nmol/L for urea; 0.006 nmol/L for estradiol; and 0.004 nmol/L for ATP) and linear range of 0.07–3.33 nmol/L for urea; 0.03–3.333 nmol/L for estradiol; 0.01–0.87 nmol/L for ATP [93] (Table 2).

Additionally, a system utilising MXeneTi_3_C_2_ nanosheet-loaded AuNCs was developed, leveraging their excellent catalytic properties for the detection of the pesticide isocarbophos. This approach combined a dual-mode nanocatalytic indicator reaction with an aptamer reaction [92]. The incorporation of AuNCs onto MXeneTi_3_C_2_ nanosheets, a two-dimensional (2D) material composed of carbides, carbonitrides and nitrides, led to an increase in surface electrons and active sites. This enhancement resulted in improved catalysis of AuNCs for AuNP production. This synergistic effect resulted in strong SERS/RRS signals, exhibiting an LoD of 4.5 × 10^−5^ nmol/L with a linear range of 1.0 × 10^−3^–2.5 × 10^−2^ nmol/L (Table 2).

AuNCs with enhanced properties were synthesised by creating rigid host–guest complexes with 6-aza-2-thiothymine and L-arginine around the AuNCs [86] (Figure 3B). The addition of poly (diallyldimethylammonium chloride) (PDDA), a positively charged, water-soluble cationic polymer, induced aggregation of the AuNPs by neutralising the charge and disrupting the citrate protective layer. In this method, PAA@Arg@ATT-AuNCs served as a signalling probe, a T-2 aptamer as the recognition element and AuNPs as a quencher. In the absence of T-2 toxins, the aptamer and PDDA form a duplex, resulting in the quenching of the fluorescence of the AuNCs. However, in the presence of T-2 toxins, the aptamer strongly binds to the toxins, causing the free PDDA to facilitate AuNP aggregation. This aggregation causes a loss of quenching capability and restores fluorescence in the AuNCs (Table 2).

NCs can also catalyse the oxidation of tetramethylbenzidine (TMB) by H_2_O_2_ and have been used for the colourimetric detection of bacteria. Thiol-modified aptamers were covalently attached to AuNCs and used to detect *Salmonella typhimurium* [91] (Figure 3C). In the presence of bacteria, they could simultaneously bind to both the aptamer@AuNCs and TMB. This proximity facilitated a strong interaction, leading to increased peroxidase-like activity towards TMB. Such enhanced activity is indicative of the sensitivity and specificity of the system, which contributed to a detection limit of 1 CFU/mL and a linear detection range of 10^1^ to 10^6^ CFU/mL (Table 2).

Aptamer-functionalised NCs bind weakly to a quencher via van der Waals interactions, enabling FRET and reducing fluorescence. This property was used to develop an AuNC-based aptasensor for detecting aflatoxin B1 and zearalenone (ZEN) [63] (Figure 3D). Au ions were mixed with L-proline and bovine serum albumin to produce blue- and red-emitting AuNCs that did not exhibit overlapping spectra when excited by a single wavelength. These AuNCs were then attached to WS_2_ nanosheets, resulting in NC fluorescence quenching. When exposed to mycotoxins, the binding preference of the aptamer prompted the release of NCs from the nanosheets, restoring the fluorescence (Table 2).

Additionally, a living bacterial cell-detecting NC biosensor was developed [80]. A AuCu bimetallic NC was produced by incorporating a polyetherimide template and functionalising it with a carboxylated aptamer. The resulting NCs can weakly interact with polydopamine nanospheres, each of which acts as an electron donor and acceptor, generating FRET. The aptamer bound to the target, leading to the detachment of the polydopamine nanosphere quenchers, thus restoring the fluorescence of the NCs. Subsequently, endonuclease (cryonase) was introduced to digest the aptamer, resulting in the release of the target and the emission of PEI-AgCu. The released target could then rebind to another aptamer, generating target cycle signal amplification. The sensor design enables the detection of *Salmonella typhimurium* down to a detection limit of 3.8 CFU/mL with a linear detection range of 10^2^–10^7^ CFU/mL (Table 2).

### 3.2. Signal Changes in NCs Produced by Aptamer-Linked DNA Templates

This system typically relies on the interaction between the aptamer and the target to initiate a structural change in the scaffold, fused form of aptamer and NC nucleation template, thereby resulting in enhancement or reduction in fluorescence emission [59,64,65,73,97,98,99,100,101,102,103,104,105,112]. Using this strategy, the AgNC template was linked with K^+^ aptamer to produce fluorescence-emitting AgNCs, enabling the measurement of vitreous K^+^ concentration for postmortem interval estimation [104]. Structural changes in the G-rich aptamer sequence resulted in fluorescence changes by influencing the C-rich AgNC motif, leading to reduced emission. This method allowed for the fast and accurate detection of vitreous K^+^ concentration, thereby facilitating the estimation of the postmortem interval with an LoD of 0.06 nM and a linear range of 0.1 nM to 1 mM (Table 3).

**Figure 4:**
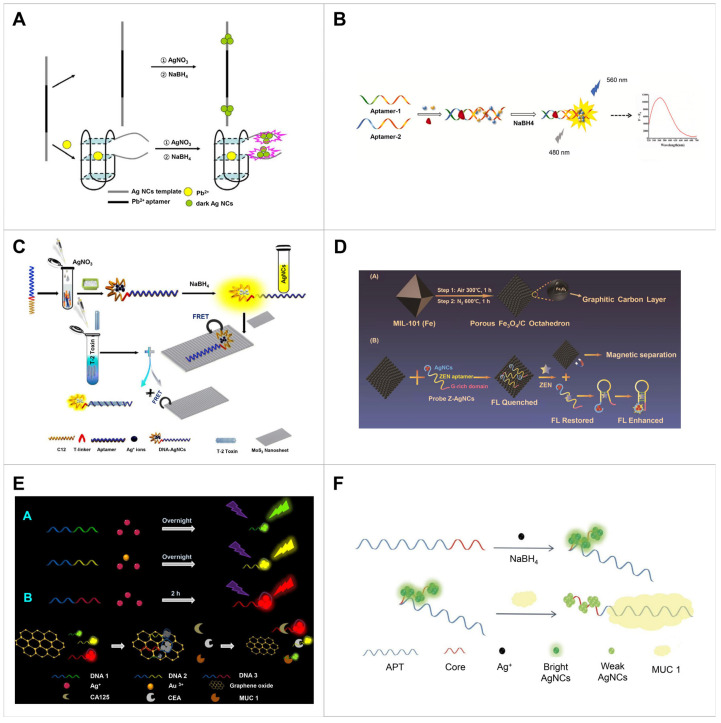
Sensing strategy based on signal changes in metal NC produced by aptamer-linked DNA template. (**A**) Detection of Pb^2+^ using a scaffold of the AgNC formation template fused with aptamer to form G-quadruplex structure in the presence of target. Reproduced with permission from [99]. Copyright 2018, Elsevier. (**B**) Detection of kanamycin using the scaffolds consisting of two split aptamer and Cu/Ag bimetal NC formation templates. Reproduced with permission from [103]. Copyright 2022, Elsevier. (**C**) Detection of T-2 toxin using a scaffold containing an aptamer, a T-linker and an AgNC template. This system also used FRET between MoS_2_ nanosheets (fluorescence acceptor) and the aptamer–AgNCs (fluorescence donor). Reproduced with permission from [64]. Copyright 2018, Elsevier. (**D**) Detection of ZEN using a scaffold consisting of an AgNC template, an aptamer and a G-rich domain. This system uses of FRET between the aptamer–AgNCs and porous Fe_3_O_4_/C acting on quenching of fluorescence and the easy separation. Reproduced with permission from [100]. Copyright 2021, Elsevier. (**E**) Detection of three different tumour biomarkers (mucin 1, carcinoembryonic antigen and cancer antigen 125), using a scaffold consisting of the same NC nucleation sequence and different aptamer sequences exhibiting different emission wavelengths for the detection of three molecules. This system used FRET between Ag/Au bimetallic NCs (donor) and GOx nanosheets (quencher). Reproduced with permission from [59]. Copyright 2018, Elsevier. (**F**) Detection of MUC1 using a scaffold consisting of C-rich template and aptamer with G-rich sequence at the end. Reproduced with permission from [112]. Copyright 2019, Elsevier. NC, nanocluster; CA125, cancer antigen 125; CEA, carcinoembryonic antigen; MUC1, mucin 1; APT, aptamer.

Additionally, an AgNC-based Pb^2+^ detecting system was developed [99] (Figure 4A). The DNA scaffold was designed to incorporate a Pb^2+^ aptamer in the middle and serve as a template for NC formation at both ends. This system adopted two distinct characteristics: one involves the formation of a Pb^2+^ binding-induced G-quadruplex [113], while the other involves enhanced fluorescence by two darkish AgNCs located nearby [114]. In the absence of the target molecule, the DNA scaffold remains linear, and AgNC formation occurs at both ends of the scaffold, resulting in diminished fluorescence intensity. Exposure to the target induces a transition of scaffold to a G-quadruplex, bringing the two darkened AgNCs bound to both ends of the scaffold in close proximity and resulting in strong fluorescence emission. This system demonstrated an LoD of 3 nM and a linear range of 5 to 50 nM (Table 3).

To amplify the fluorescence intensity of the sensing system, two split aptamers were used and applied to detect kanamycin [103] (Figure 4B). Cu^2^⁺ and Ag⁺ ions were introduced to initiate a dark reduction. Subsequently, the reducing agent NaBH₄ was added, resulting in the formation of Cu/AgNCs, along with a poor fluorescent signal. However, in the presence of kanamycin, the interaction between split aptamers was facilitated, owing to their inherent affinity for the target. This interaction caused a significant enhancement of the fluorescent signal of NCs (Table 3).

This strategy uses a scaffold integrated with the aptamer to modify both its affinity for the target and the optical properties of the NCs. To address limitations, aptamers with high affinity for NCs were used. A tetracycline-detecting AgNC aptasensor was developed, where the tetracycline-specific aptamer, rich in cytosine and capable of forming a G-quadruplex, also serves as a template for AgNC nucleation [97]. Upon target binding, the aptamer undergoes a structural transformation into a hairpin, causing aggregation of the fluorescent AgNCs, which increases size and reduces fluorescence (Table 3).

Another strategy involves the use of a quencher, where the fluorescence emission can be switched between ‘turn-on’ and ‘turn-off’ modes through the competition between the targets and quenchers. NCs have been combined with nanomaterials, such as GOx [60], Mo_2_C nanotubes [61], CNPs [62], WS_2_ nanosheets [63] and MoS_2_ nanosheets [56], which act as a fluorescence acceptor. The coupling of these materials induces fluorescence resonance FRET. Using this mechanism, a system for detecting T-2 toxin was developed [64] (Figure 4C). The DNA template was designed by linking sequences for AgNC nucleation to a T-2 toxin-specific aptamer, with the addition of a T5 spacer to account for steric hindrance. Upon the deposition of the AgNCs on the MoS_2_ sheet, the aptamer was adsorbed on the MoS_2_ surface through van der Waals force. This interaction resulted in FRET facilitated by the *sp*^2^ hybrid crystal domain, ultimately resulting in fluorescence quenching. The presence of T-2 toxin in the sample strongly causes competitive binding with aptamer, leading to AgNC separation from the MoS_2_ sheet and subsequent fluorescence recovery in the NCs. The aptamer binds specifically to the T-2 toxin on the Mo_2_S sheet. The significant quenching effect of MoS_2_ contributed to an LoD of 0.93 pg/mL and a linear range of 0.005–500 ng/mL (Table 3).

A scaffold containing a G-rich domain was developed for a ‘turn-on’ NC-based aptasensor for fluorescence enhancement [100] (Figure 4D). The scaffold was specifically designed to include an AgNC template, an aptamer and a G-rich domain. This sensor used FRET between the aptamer–AgNCs and a porous Fe_3_O_4_/carbon material, which served to quench the fluorescence and facilitate easy separation of the components. Upon binding the aptamer–AgNCs to the target, the complex was released from the Fe_3_O_4_/C material. The conformational change of the aptamer results in the NCs being closer to the G-rich domain of the scaffold, leading to enhanced fluorescence. This system could detect ZEN up to 2 × 10^−3^ ng/mL, with a dynamic range of 0.01 ng/mL to 250 ng/mL (Table 3).

Furthermore, the aptamer sequences linked to the NC template can change the emitted wavelength of NCs, allowing for the detection of multiple target molecules by altering the aptamer sequence while retaining the NC template. Leveraging this property, an Ag/Au bimetallic NC-based biosensor was developed for the detection of three tumour biomarkers, mucin 1 (MUC1), carcinoembryonic antigen and cancer antigen 125 [59] (Figure 4E; Table 3).

Although stability at high salt concentration is suitable for the dsDNA formation reaction-mediated biosensing strategy [74,75], the low efficiency of synthesis of dsDNA reduces CuNC production yield, thereby resulting in weak fluorescence. The sensitivity of a biosensing system can be improved by incorporating a signal amplification reaction [115]. For example, the synthesis of dsDNA-templated CuNCs was combined with the target-cycling strand displacement amplification for the detection of adenosine triphosphate (ATP) [73]. The scaffold was designed with an AT-rich hairpin stem for CuNC formation and an aptamer for ATP binding. In the absence of ATP, no binding occurs, and DNA polymerase does not extend the AT-rich region. Upon ATP binding, the scaffold undergoes a conformational change, enabling polymerase to extend the AT-rich region, releasing ATP from the aptamer and binding it to another scaffold. This target recycling amplifies the scaffold and boosts CuNP production and blue fluorescence. This system exhibited an LoD of 5 pM with a linear range of 0.01 to 10 nM (Table 3).

The NC aptasensor was used for in vivo imaging and in vitro detection [112] (Figure 4F). To detect the tumour marker, MUC1, an AgNC scaffold was designed with a C-rich template and an aptamer with a G-rich sequence at the end, enhancing the fluorescence of the DNA–AgNCs [116]. Upon binding to the target, the aptamer detached from the florescence-emitting AgNC surface, resulting in a decrease in the fluorescence of the AgNCs (Table 3).

### 3.3. Signal Changes in NCs Induced by Aptamer–DNA Template Hybridisation

The hybridisation-induced signal-switching system consists of two strands: the first strand serves as a template-extending strand and contains a sequence essential for the formation of NCs and a partially complementary sequence to the aptamer. The second strand comprises the aptamer, which functions as both a signal transducer and a quencher. NCs are generated from the template in the region complementary to the aptamer, with their process being controlled by the aptamers through hybridisation; this results in a shift in fluorescence.

**Figure 5:**
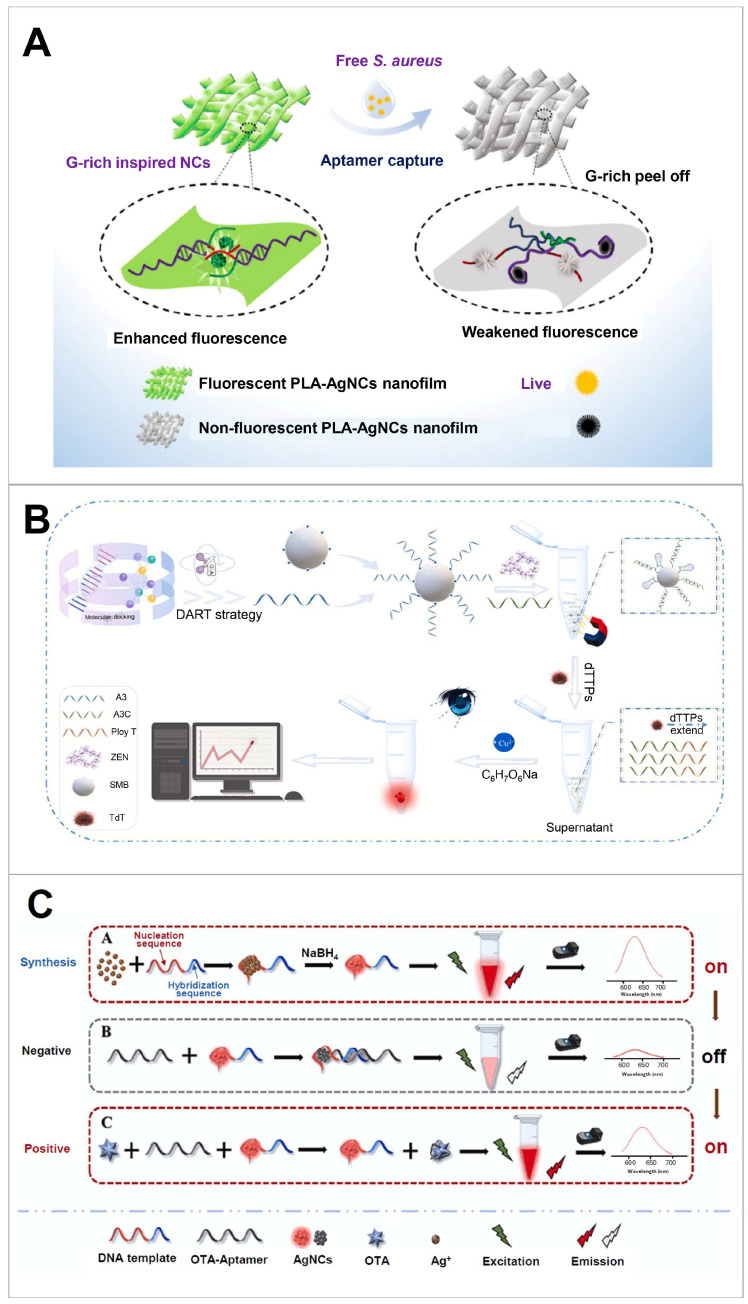
Sensing strategy based on signal changes in metal NCs induced by aptamer–DNA template hybridisation. (**A**) Detection of two different bacterial cells (*Staphylococcus aureus* and *Escherichia coli*) using AgNC bound with hybrid DNA of NC scaffold and bacteria-specific aptamer. This system used the antibacterial effect of AgNC and enhanced AgNC fluorescence via electrospinning to PLA, forming nanofilms. Reproduced with permission from [107]. Copyright 2021, American Chemical Society. (**B**) Detection of ZEN using dual-signal amplification mechanism based on TdT amplification and CuNC fluorescence enhancement. Reproduced with permission from [76]. Copyright 2024, Elsevier. (**C**) Detection of ochratoxin A using aptamer serving as both the recognition and quenching reagent. This system used scaffold sequences screened for emitting or quenching fluorescence. Reproduced with permission from [106]. Copyright 2023, Elsevier. NC, nanocluster; PLA, polylactic acid; ZEN, zearalenone; SMB, streptavidin-coated magnetic bead; TdT, terminal deoxynucleotidyl transferase; OTA, ochratoxin A.

A pathogenic bacteria-detecting NC aptasensor was developed using this strategy [107] (Figure 5A). The DNA template included a nucleation region for AgNC and a region partially complementary to the aptamer. The aptamer-linked G-rich sequence formed a duplex with the template, promoting AgNC nucleation at the G-rich region and enhancing fluorescence. Electrospinning with polylactic acid improved AgNC’s surface area, biodegradability and biocompatibility, boosting their antibacterial activity. In the presence of target bacteria, the aptamers captured the bacteria, causing conformational changes and detachment from the duplex, which reduced fluorescence. The aptamer then bound to the bacteria, leading to their elimination upon contact with the AgNC. This AgNC exhibited a linear relation range of 10^7^ to 10^11^ CFU/mL with fluorescence intensity (Table 4).

Since the affinity of the aptamer against the target determines the sensitivity and specificity of the sensing system, an in silico molecular docking program-based strategy was also employed [76] (Figure 5B). The aptamer design for ZEN involved predicting the key binding sites via the program. In a one-pot reaction, a biotinylated aptamer was attached to streptavidin-coated magnetic beads, and the aptamer formed a duplex with an added oligo that was complementary to the aptamer. Upon exposure to ZEN, the oligos detached from the aptamer and underwent modification to extend poly-T tails at 3′-end through a reaction assisted by terminal deoxynucleotidyl transferase. Subsequently, CuNCs were formed at the T-rich region after adding a reducing agent owing to the high affinity of Cu ions for T bases; this generated red fluorescence. The sensor exhibited an LoD of 0.1 ng/mL with a linear range of 10^−1^ ng/mL to 10^3^ ng/mL (Table 4).

Aptamers can serve as both recognition and quenching reagents, eliminating the need for additional quenchers. Leveraging this property, an ochratoxin A (OTA)-detecting AgNC aptasensor was developed [106] (Figure 5C). Red-emitting AgNCs were formed by mixing with a scaffold consisting of a C-rich template, a linker region and a region partially complementary to the OTA-specific aptamer. Different sequences in the hybridisation region can change the fluorescence of AgNCs, even when using the same template for nucleation [49,117]. The hybridisation sequences responsible for either emitting or quenching fluorescence were identified through screening. In the absence of the target, the scaffold was hybridised with the aptamer, which acted as a quencher, thereby decreasing the fluorescence. Conversely, in the presence of the target in the sample, they attached to the aptamer, resulting in red-emitting AgNCs showing no change. This method contributed to an LoD of 1.3 nM with a linear range of 10 to 125 nM, requiring no labelling, additional quenchers or amplification processes (Table 4).

## 4. Conclusions and Future Perspectives

Aptamer-coupled metal NC-based sensing systems offer advantageous properties, including programmability, biocompatibility and molecular recognition. This review summarises recent advancements in metal NC-based aptasensor for the detection and/or imaging of molecules and chemicals, with a focus on the role of aptamers in modulating the optical signal emission of metal NC-based sensing system. Many strategies have been suggested to enhance the performance of NC-based aptasensors, with ongoing efforts directed towards developing highly sensitive, specific, multiplexed, fast and cost-effective assays. These strategies have been discussed in this review. These examples confirm that optoelectronic sensing systems are a rapidly developing technology with advantages, such as facile synthesis, label-free detection, low cost and simple operation.

Despite these advantages, there is still a need to improve the sensing performance of NC-based systems to enhance their capability. A major challenge in this field is the precise regulation of NC properties, such as the colour, brightness and enhancement ratio. The programmability of DNA templates for NC nucleation offers a way to modulate their properties by altering the base sequence and length. However, the intricate relationship between the DNA sequence and the resulting NC colour presents a hurdle, complicating the design of DNA templates and limiting the widespread applicability of NCs in diverse. Additionally, achieving precise synthesis with well-defined atomic structures remains challenging, as the mechanisms linking NC formation, structure and properties are not fully understood. While phenomena like aggregation-induced emission (AIE) improve quantum yields, their complexity requires further investigation. Stability issues during storage and transport also restrict the practical applications of NCs, and research on advanced nanocomposites still remains. In practical application, the complexity of substrates and preprocessing requirements confines applications to simpler matrices like water [118,119]. Scalability poses another challenge due to reliance on expensive noble metals (e.g., Au, Ag and Pt), high production costs and limited exploration of polymetallic systems. These constraints underscore the need for cost-effective, stable and broadly applicable NC systems to advance their diagnostic and sensing capabilities for real-world applications.

To address these limitations, the development of screening platforms and prediction programs may provide valuable tools for optimising NC properties and expanding their usability in diverse fields. For example, the programmable colour of NCs can be achieved using an activatable fluorescent probe, NanoCluster Beacon (NCB), which involves hybridisation with a scaffold, [50,120,121]. NCBs offer a range of activation colours originating from a dark state (not via FRET), resulting in fluorescence enhancement ratios ranging from 1500- to 2400-fold [122,123]. A recent study reported the use of a next-generation sequencing chip screening platform for the selection of NCBs, identifying a critical zone within the activator (positions 7–12) that stabilises bright AgNC chromophores. This was achieved through a high-throughput screening of over 40,000 activators [124]. Furthermore, the screening results were analysed and used to design bright and multi-colour NCBs by employing machine learning algorithms. The sequence-based models may present challenges in predicting AgNC-DNA properties, limiting their utility for imaging and sensing applications that require control over multiple properties, such as emission colour, brightness, chemical stability and sensitivity to analytes.

A pertinent challenge in the advancement of machine learning-enabled design methods for emerging material systems is the lack of fundamental knowledge. This limitation has spurred the development of a multi-objective model for AgNC-DNA design using a regularised VAE that automates feature extraction and effectively handles imbalanced data without requiring domain expertise [125]. This model successfully generated DNA sequences for bright green and rare NIR AgNC-DNAs, enhancing their relative abundance by 3.7 and 4.9 times, respectively, while improving emission brightness compared to the training data. The model, supported by Shapley value analysis, reveals critical insights into the significance of nucleobase patterns in shaping AgNC-DNA properties. These models can be adapted for various sequence-based biomolecules, including protein and peptide materials.

The use of machine learning in template design holds significant potential, not only for nanocluster (NC) templates but also for the development of aptamers. Traditionally, aptamer development has relied on the SELEX (Systematic Evolution of Ligands by Exponential Enrichment) method, which is time-consuming, has low success rates and produces a limited range of aptamer candidates [126]. In contrast, integrating machine learning with evolving computational technologies offers a promising solution to overcome these limitations and significantly enhance the aptamer design and optimisation process [127]. One of the key advantages of machine learning in aptamer development is its ability to simultaneously predict multiple properties of aptamers. For instance, machine learning can be used to design aptamers based on affinity and specificity or to improve existing aptamer sequences by enhancing properties such as nuclease stability and dissociation kinetics [128,129]. Furthermore, artificial intelligence, including machine learning and deep learning algorithms, has already been widely applied in the research and development of new drugs and target molecules, demonstrating its potential to revolutionize aptamer development as well.

In addition to template diversity, the use of FRET has enhanced the properties of NCs. However, the constraints associated with a limited number of FRET pairs and increased costs have propelled the development of quencher-free systems. A recent study introduced a non-FRET reporter, known as the Subak reporter, designed for altering AgNC colours [130]. The Subak reporter exhibits a nuclease-based fragmentation-induced colour-switching property that uses the altered base-cluster interacting footprint and the changed size/shape of the fragmented AgNC. It can provide a low-cost, non-FRET probe with ratiometric sensing capability and facilitate biosensing performance when combined with RNA-cleaving DNAzymes and RNA-targeting Cas effectors.

In parallel, biosensors coupled with aptamers have been increasingly developed to exploit the unique physical and chemical properties, along with the excellent biocompatibility, of MXene—a material composed of metal and carbon. MXene-based aptasensors are gaining traction as versatile devices for various applications, including cancer biomarker detection, food safety assessment and environmental monitoring. Notably, MXene’s high electrical conductivity and large surface area offer an innovative platform for biological molecule recognition, paving the way for next-generation diagnostic and analytical tools [92,131]. Therefore, combining MXene with metal NCs holds the potential to further enhance the properties of NC-based systems.

With ongoing advances in this field, we believe that metal NC-based aptasensing systems will emerge as a promising option for monitoring and detecting diverse molecules in food, water and environmental sources during production processes.

## Figures and Tables

**Table 1:** Advantages and limitations of metal NCs in biological applications ^a^.

Metal	Advantages	Limitations
AuNC	Lower toxicity than many other metals Strong, size-tunable fluorescence Less prone to oxidation, ensuring consistent performance Presence of ECL luminophore with tunable luminescence	High cost High size dependence and the need for precise control over size and shape for fluorescence The need for a strict design of DNA template owing to unique DNA sequence dependency Low stability at high salt concentrations
AgNC	Strong fluorescence and photoluminescence Diverse fluorescence colours ranging from blue/green to NIR Antimicrobial properties Low toxicity Excellent stability	Toxicity to cells and organisms Higher susceptibility to oxidation and instability compared to AuNCs, which may affect their performance over time High complexity of the sequence-to-colour rules and DNA template design Low stability at high salt concentrations
CuNC	Abundance, cost-effectiveness and availability of precursor for construction Rapid synthesis (several minutes) Long Stokes shift favourable for minimising the interference from background signals in biological systems.	Susceptibility to oxidation and instability Limited fluorescence colours Relatively low stability
Bimetallic NC	Enhanced optical, catalytic and stable properties: synergistic properties from combined metals Customisable functions achieved by varying the composition and ratio of the metals used	Complex synthesis Inter-metal interactions, which lead to unpredictable or non-ideal properties, thus requiring careful optimisation

^a^ Abbreviations: NC, nanocluster; ECL, electrochemiluminescence.

**Table 2:** Examples of metal NC-based strategies utilising signal emission changes in aptamer-linked metal NCs ^a^.

Metal	Usage	Analyte	Detection Method	Linear Range	LoD	Sample	Feature	Reference
Au	Detection	Kanamycin	Electrochemiluminescence	50.00 fg/mL–50.00 ng/mL	32.90 fg/mL	Milk, honey	Use of cucurbit[7]uril@Try-MPA-AuNC with improved ECL performance as the anode signal probe.	[47]
Au	Detection	Aflatoxin B1 (AFB1), zearalenone (ZEN)	Fluorescence	0.005–100 ng/mL	0.34 pg/mL for AFB1; ZEN: 0.53 pg/mL for ZEN	Maize	Production of blue- and red-emitting AuNCs for dual-colour simultaneous detection by combining with L-proline and bovine serum albumin. Use of FRET between the AuNCs and WS_2_ quencher.	[63]
Au	Detection	Mycotoxin (patulin)	Fluorescence	0.01–100 ng/mL	8.5 ng/L	Apple and grape juice	Use of FRET between aptamer@AuNCs (acceptor) and BSA@MnO_2_ nanoflakes (quencher).	[81]
Au	Detection	Cocaine	Electrochemistry	0.001–1.0 ng/mL	Electrochemical impedance spectroscopy, 1.29 pM; differential pulse voltammetry, 2.22 pM	Human serum, urine, saliva	Use of AuNCs@Zr-MOF-based nanosheets.	[82]
Au	Detection	Estradiol	SERS/RRS	0.333–5.33 nmol/L (SERS); 0.33–4.00 nmol/L (RRS)	0.150 nmol/L (SERS); 0.23 nmol/L (RRS)	Urine	Dual-mode SERS and RRS aptasensor. Use of AuNC-loaded COF catalyst. Based on the Apt modulating AuBtPD catalysis with AuNP indicator in the presence of a VB4R molecular probe.	[83]
Au	Detection	AFB1	Fluorescence/colourimetry	5–400 ng/mL for fluorescence; 20–400 ng/mL for colourimetry	1.91 ng/mL; 12.16 ng/mL for colourimetry	Wheat	Turn-on dual-mode FRET aptasensor. Use of Arg/ATT-AuNCs (donor) and AgNPs (quencher).	[84]
Au	Detection	Tetracycline	Colourimetry	1–16 μM	46 nM	Drug, milk	Use of peroxidase-like AuNC.	[85]
Au	Detection	Mycotoxin (T-2)	Fluorescence	0.001−100 ng/mL	0.57 pg/mL	Maize	Use of the green-emitting AuNCs synthesised by employing rigid host–guest assemblages between ATT and Arg around the AuNCs. Use of FRET between PAA@Arg@ATT-AuNCs (fluorescence donor) and AuNPs (energy receptor).	[86]
Au	Detection	Staphylococcal enterotoxin B (SEB)	Colourimetry	1–700 ng/mL	1.0 × 10^−12^ g/mL	Corn, rice, flour	Use of peroxidase-like AuNC-chitosan composite membrane.	[87]
Au	Detection	Kanamycin	Fluorescence	0.04 nM–7.0 nM	0.032 nM	Milk	Use of BSA-attached AuNC.	[88]
Au	Detection	Vancomycin	Fluorescence	0.01–100 μg/mL	2.79 ng/mL	Serum, rabbit	Dual-emission biosensor. Use of blue-emitting aggregation-induced emission luminogens and aptamer-modified red-emitting AuNCs–aptamer.	[89]
Au	Detection	Adenosine	Electrochemistry	0.1 nM–1 mM	0.1 nM	Mouse	Real-time target monitoring in vivo. Use of rGOx-AuNC-modified electrode surface.	[90]
Au	Detection	*Salmonella typhimurium*	Colourimetry	10^1^–10^6^ CFU/mL	1 CFU/mL	Eggshell, egg white	Enabling simultaneous binding of bacteria to both the aptamer@AuNCs and TMB, facilitating peroxidase-like activity due to the increased proximity of these interactions.	[91]
Au	Detection	Pesticide isocarbophos	SERS/RRS	1.0 × 10^−3^–2.5 × 10^−2^ nmol/L	4.5 × 10^−5^ nmol/L	Farmland water	Use of MXene-loaded AuNC catalyst. Dual-mode nanocatalytic indicator reaction with aptamer reaction.	[92]
Au	Detection	Urea, estradiol, ATP	SERS/RRS	0.07–3.33 nmol/L for urea; 0.03–3.333 nmol/L for estradiol; 0.01–0.87 nmol/L for ATP	0.07 nmol/L for urea; 0.006 nmol/L for estradiol; 0.004 nmol/L for ATP	Urine	Dual-mode SERS and RRS aptasensor. Use of an AuNC-doped COF catalyst.	[93]
Cu	Detection	Oxytetracycline	SERS, RRS	SERS, 37.5–300 ng/L; RRS, 37.5–225 ng/L	SERS, 18.0 ng/L; RRS, 25.0 ng/L	Water	CuNC synthesis under a reduction solution of L-cysteine and NaOH. Use of CuNC catalyst for an AuNP generation reaction.	[95]
Au+Cu bimetal	Detection	Hg^2+^	Fluorescence	0.1–9.0 μM	4.92 nM	Porphyra	Use of aptamer-modified AuCu bimetallic NCs, which remained well dispersed in the solution without Hg^2^⁺ but aggregated upon Hg^2^⁺ addition to form a T–Hg–T structure, resulting in altered fluorescence intensities due to FRET and visible changes in fluorescent colour.	[96]
Au+Cu bimetal	Detection	DON	Fluorescence	5–100 ng/mL	1.87 ng/mL	Maize flour	FRET-based aptasensor using AuCu bimetallic NCs (donor) and MoS_2_ nanosheets (quencher). Attachment of NC with a thiol-modified aptamer.	[94]
Ag+Cu bimetal	Detection	*Salmonella typhimurium*	Fluorescence	10^2^–10^7^ CFU/mL	3.8 CFU/mL	Milk, orange juice, chicken, egg white	NC formation by adding PEI as the polymer template. FRET-based aptasensor using aptamer-attached PEI-AgCu bimetallic NCs (donor) and polydopamine nanospheres (quencher). Combination with a cryonase-based signal amplification method, which splits PEI-AgCu from the aptamer; the release target can repeatedly bind to another aptamer, thereby emitting fluorescence. Cryonase-assisted target cycle signal amplification.	[80]

^a^ Abbreviations: LoD, limit of detection; NC, nanocluster; SERS, surface-enhanced Raman scattering; RRS, resonance Rayleigh scattering; MPA, mercaptopropionic acid; PAA, polyacrylic acid; COF, covalent-organic framework; FRET, fluorescence resonance energy transfer; ATT, 6-aza-2-thiothymine; PEI, polyethylenimine; GOx, graphene oxide; rGOx, reduced GO; BSA, bovine serum albumin.

**Table 3:** Examples of metal NC-based strategies utilising signal changes in aptamer-linked NCs ^a^.

Metal	Usage	Analyte	Detection Method	Linear Range	LoD	Sample	Feature	Reference
Ag	Detection/imaging	Mucin1 (MUC1)	Fluorescence	0.1–100 nM	0.05 nM	MCF-7 cell	Use of scaffold consisting of C-rich template and aptamer with G-rich sequence at the end.	[112]
Ag	Detection	Potassium ion (K^+^)	Fluorescence	0.1 nM–1 mM	0.06 nM	Vitreous humour	Use of a guanine quartet potassium aptamer sequence and a C12 AgNC sequence. Structural changes in the G-rich aptamer sequence, driving fluorescence changes by simply affecting the C-rich AgNC motif.	[104]
Ag	Detection/antimicrobial activity	*Pseudomonas aeruginosa*	Fluorescence	ND	ND	*Galleria mellonella* larvae	Use of a scaffold to form both the aptamer and the NC-generating region. Antimicrobial activity testing in vitro and in an in vivo animal model.	[65]
Ag	Detection	Tetracycline (TET)	Fluorescence	20 ng/mL–10 g/mL	11.46 ng/mL	Milk	Use of aptamer sequence of TET rich in cytosine and capable of forming a G-quadruplex structure, which also serves as a template for AgNC nucleation.	[97]
Ag	Detection	Staphylococcal enterotoxin A (SEA)	Fluorescence	0.5–1000 ng/mL	0.3393 ng/mL	Milk	Fluorescence quenching by binding to ssDNA aptamer/AgNC using polypyrrole NPs (PPyNPs) and a quencher. Use of the competitive binding interaction between SEA, PyNPs and the aptamer.	[98]
Ag	Detection	Pb^2+^	Fluorescence	5–50 nM	3.0 nM	Lake water, tap water	Use of a scaffold with a G-quadruplex aptamer specific for Pb^2+^ and AgNC templates at both ends. Use of enhanced fluorescence properties by two dark-coloured AgNCs nearby	[99]
Ag	Detection	T-2 toxin (Fusarium mycotoxin)	Fluorescence	0.005–500 ng/mL	0.93 pg/mL	Maize, wheat	Use of a template containing an aptamer and an NC scaffold. Use of FRET between MoS_2_ nanosheets (fluorescence acceptor) and the aptamer-AgNCs (energy donor).	[64]
Ag	Detection	ZEN	Fluorescence	0.01–250 ng/mL	2 × 10^−3^ ng/mL	Maize, wheat	Turn-on FRET aptasensor. Use of a scaffold consisting of an AgNC template, an aptamer and a G-rich domain. Use of FRET between the aptamer/AgNCs and porous Fe_3_O_4_/C. Fe_3_O_4_/C acting on quenching of fluorescence and the easy separation. G-rich domain for fluorescence enhancement. Thirty-day stability.	[100]
Ag	Detection	*Staphylococcus aureus*	Electrochemistry	10^1^–10^6^ CFU/mL	1.0 CFU/mL	Tap water, river water	Detection of *Staphylococcus aureus* using an aptamer-based sandwich assay.	[101]
Ag	Detection	Organic mercury	Fluorescence	0.05–2.0 μM	5.0 nM	Water, fish muscle	Use of scaffold consisting of an AgNC template and an organic mercury-recognizing T-rich sequence.	[105]
Cu	Detection	ATP	Fluorescence	0.01–10 nM	5 pM	None	Use of a scaffold consisting of a hairpin stem containing an AT-rich sequence for the formation of a CuNC and an aptamer for ATP binding. Combination of dsDNA-templated CuNCs synthesis with the target-cycling strand displacement amplification for signal amplification.	[73]
Au+Ag bimetal	Detection	MUC1, carcinoembryonic antigen, cancer antigen 125	Fluorescence	1.33–200 ng/mL for MUC1; 6.7 ng/mL–13.3 ug/mL for carcinoembryonic antigen; 2 ng/mL–6.7 ug/mL for cancer antigen 125	0.18 ng/mL for MUC1; 3.18 ng/mL for carcinoembryonic antigen; 1.26 ng/mL for cancer antigen 125	Human serum	Use of a scaffold consisting of the same NC nucleation sequence and different aptamer sequences, exhibiting different emission wavelengths for the detection of three molecules. FRET-based aptasensor using AuAg bimetallic NCs (donor) and GOx nanosheets (quencher).	[59]
Ag+Cu bimetal	Detection	Kanamycin	Fluorescence	80 nM–10 μM	13.3 nM	Tap water, milk	Combination of two split kanamycin aptamers, adding Cu^2^⁺ and Ag⁺ for a dark reaction, followed by the reducing agent NaBH₄ to form AgCu bimetallic NPs, which produced a weak fluorescent signal that was significantly enhanced in the presence of kanamycin due to the affinity of the aptamers for each other.	[103]
Ag+Pt bimetal	Detection	Thrombin	Colourimetry	1–50 nM	2.6 nM	Human thrombin	Use of co-synthesised bimetallic NCs produced in a DNA template. Use of good peroxidase-like catalytic activity of Pt NC deposited by the galvanic replacement reaction between Ag(0) and Pt(II) on the surface of the AgNCs.	[102]

^a^ Abbreviations: LoD, limit of detection; NC, nanocluster; FRET, fluorescence resonance energy transfer; GOx, graphene oxide; ND, not determined.

**Table 4:** Examples of metal NC-based strategies utilising signal changes induced by aptamer–DNA template hybridisation ^a^.

Metal	Usage	Analyte	Detection Method	Linear Range	LoD	Sample	Feature	Reference
Au	Detection	Deoxynivalenol	Fluorescence/SERS	0.1–100 ng/mL	Fluorescence, 0.08 ng/mL; SERS, 0.06 ng/ml	Wheat flour	Dual-mode aptasensor. Selection of complementary DNA-modified Au NCs as a fluorescence probe. Use of TAMRA as a Raman label. Use of aptamer-modified Ag NPs/MPDA as the SERS substrate and fluorescence quencher.	[109]
Ag	Detection	Ochratoxin A (OTA)	Fluorescence	10–125 nM	1.3 nM	Maize, wheat	Turn-on FRET aptasensor. Use of aptamer serving as both the recognition and quenching reagent. Screening of scaffold sequences for emitting or quenching fluorescence. Detection time of 45 min.	[106]
Ag	Detection/antimicrobial activity	*Staphylococcus aureus*, *Escherichia coli*	Fluorescence	1 × 10^7^–1 × 10^11^ CFU/mL	ND	Milk	Use of AgNC bound with hybrid DNA of NC scaffold and bacteria-specific aptamer. Use of the antibacterial effect of AgNC. Enhanced AgNC fluorescence via electrospinning to PLA, forming nanofilms.	[107]
Ag	Detection	Adenosine	Fluorescence	0–200 μM	2.7 μM	Human serum	Use of aptamer kissing module system using loop–loop interactions. Binding of adenosine to aptamer to form a loop structure, binding via kissing interaction to an oligo with AgNC sequence and fluorescence expression due to the proximity of a G-rich overhang on the AgNC side through the binding of a complementary stem. Hybridisation-induced signal-switching system.	[108]
Cu	Detection	ZEN	Fluorescence	10^−1^–10^3^ ng/mL	0.1 ng/mL	Water	Dual-signal amplification mechanism based on TdT amplification and CuNC fluorescence enhancement	[76]

^a^ Abbreviations: LoD, limit of detection; NC, nanocluster; SERS, surface-enhanced Raman scattering; FRET, fluorescence resonance energy transfer; PLA, polylactic acid; TdT, terminal deoxynucleotidyl transferase; ND, not determined.

## Data Availability

No new data were created in this study. Data sharing is thus not applicable to this article.
